# Human mesenchymal stem cells preferentially migrate toward highly oncogenic human hepatocellular carcinoma cells with activated EpCAM signaling

**DOI:** 10.18632/oncotarget.17633

**Published:** 2017-05-05

**Authors:** Berwini Endaya, Shou P. Guan, Jennifer P. Newman, Hung Huynh, Kian C. Sia, Siao T. Chong, Catherine Y.L. Kok, Alexander Y.F. Chung, Bin B. Liu, Kam M. Hui, Paula Y.P. Lam

**Affiliations:** ^1^ Division of Cellular and Molecular Research, National Cancer Centre, Singapore City, Singapore; ^2^ Griffith Health Institute, Griffith University, Southport, Australia; ^3^ Department of General Surgery, Singapore General Hospital, Singapore City, Singapore; ^4^ Liver Cancer Institute of Fudan University, Shanghai, China; ^5^ Department of Physiology, National University of Singapore, Singapore City, Singapore; ^6^ Department of Biochemistry, National University of Singapore, Singapore City, Singapore; ^7^ Institute of Molecular and Cell Biology, A*STAR, Singapore City, Singapore; ^8^ Cancer and Stem Cells Biology Program, Duke-NUS Graduate Medical School, Singapore City, Singapore

**Keywords:** human bone marrow-derived MSC, tumor tropism, EpCAM signaling, human hepatocellular carcinoma

## Abstract

The epithelial cell adhesion molecule (EpCAM) is a type I transmembrane glycoprotein that is regarded as one of the markers for tumor initiating cells (TIC) in human hepatocellular carcinoma (HCC). Much work has been directed towards targeting these TICs as a mean of placing these master regulators of cell proliferation and drug resistance under control. Human bone marrow-derived mesenchymal stem cells are known to exhibit an innate property of tumor tropism. However, the possible relationship between MSC and TIC is not well understood. In this study, we show that MSC migration to HCC can be effectively inhibited by TACE and γ-secretase inhibitors that stop the activation of EpCAM signaling event. Silencing of EpCAM expression through siRNA and antibody approaches also resulted in impaired MSC migration. By contrast, increase levels of EpICD proteins in HCC cells and HCC mouse xenografts resulted in enhanced MSC migration. Taken together, these findings show that MSC is drawn to the more oncogenic population of HCC, and could potentially serve as a cell-based carrier of therapeutic genes to target EpICD-enriched hepatic tumor cells.

## INTRODUCTION

Hepatocellular carcinoma (HCC) is the fifth leading cause of morbidity and mortality in the world [1]. Surgical resection, percutaneous ablation and liver transplantation are standard treatments for HCC, but this is only applicable to a small proportion of patients with early tumors. Furthermore, treatment is further challenged with high tumor recurrence attributed to intrahepatic metastasis from the primary tumor. Thus, the disease warrants development of innovative therapies to bring about better controls to tumor spread and proliferation.

Human mesenchymal stem cells (MSC) have been employed as tumor-tracking missiles for cancer treatment due to their inherent biological property to migrate toward tumors or site of injury and inflammation [2]. The findings that MSC could target microscopic tumors when administered systemically [3] bear important clinical relevance to the therapeutic utility of these cells for treating intrahepatic metastases without harming the surrounding normal hepatocytes. This was recently shown when MSC modified with human pigment epithelium-derived factor could localize and efficiently suppress the spread and growth of primary and metastatic tumors [4]. Radiation has been shown to increase the expression of inflammatory mediators which resulted in enhanced MSC engraftment in the irradiated limb than in the contralateral unirradiated limb of a bilateral murine breast carcinoma mouse model [5]. Thus, we hypothesize that during cancer progression, tumor initiating cells (TIC) may be secreting higher levels of inflammatory cellular factors that favor its growth in comparison to cells with lesser oncogenic potential. As a result, enhanced MSC engraftment can be found at the site of pathological lesions.

EpCAM (also known as CD326; ESA; HEA125 and TACSTD1) is a type I transmembrane glycoprotein (40 kDa) consisting of an ectodomain, a transmembrane and an intracellular cytoplasmic domain [6]. EpCAM has been suggested as one of the prognostic markers for HCC [7] and pancreatic cancer patients [8]. It is frequently overexpressed in many human malignancies, but found at low levels in normal epithelia [9]. The extracellular domain of EpCAM contains two cysteine-rich repeats representing an EGF-like repeat and a thyroglobulin repeat domain [10], whose function has been associated with inhibiting cathepsins. The intracellular domain of EpCAM (26 amino acids) contains two binding sites for α-actinin through which the cytoplasmic domain of EpCAM can interact with the actin cytoskeleton, thus, conferring stabilization of EpCAM-mediated intercellular adhesions. EpCAM requires regulated intramembrane proteolysis (RIP) for the activation of subsequent downstream signaling event [9]. It is first cleaved by tumor necrosis-factor α converting enzyme (TACE), followed by a gamma-secretase complex comprising presenilin 2 (PS2) [11], and the extracellular domain (EpEx) is released as a soluble protein. The intracellular domain of EpCAM, also known as EpICD, enters the cell nucleus and associate with the scaffold protein four and a half-lim domain protein 2 (FHL2), β-catenin and Lef-1 which eventually leads to transcription of downstream target genes such as c-Myc, cyclin A, E and D1 [7, 12].

In the present study, we aimed to study the possible relationship between TIC and the tumor tropism of MSC. We demonstrated that MSC migration to HCC tumors can be effectively inhibited by TACE and γ-secretase inhibitors, which prevented the activation of EpCAM signaling. Silencing of EpCAM expression through siRNA and antibody approaches also resulted in impaired MSC migration. By contrast, ectopic expression of EpICD resulted in enhanced tumor cells proliferation in HCC cells via activation of c-Myc, and confers enhanced resistance against sorafenib. Taken together, we have shown that MSC is drawn to the more oncogenic population of HCC and could serve as a cell-based carrier of therapeutic genes whereby specific targeting of TIC among the heterogeneous nature of tumor mass may be made possible, which is otherwise difficult to target by conventional approaches.

## RESULTS

### Human MSC migrates to HCC tumors

Bone marrow-derived human MSC were immunopositive for CD44 and double-positive for CD90 and CD105 but negative for CD34 as determined by flow cytometry analysis (Supplementary Figure 1A). MSC also expressed the surface markers CD13, CD29 and CD73 but not CD45 (Supplementary Figure 1B). The multipotency of MSC was confirmed by culturing the cells in defined induction medium to induce differentiation into osteogenic and adipogenic lineage (Supplementary Figure 1C).

**Figure 1:**
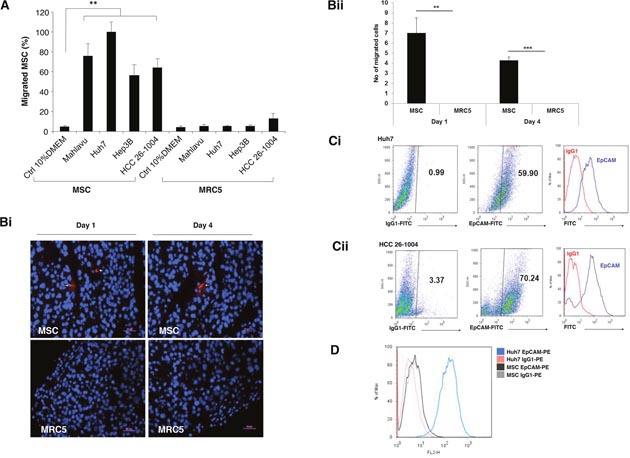
Human MSC migrates to HCC cell lines and tumors **(A)** Migration of MSC or MRC5 toward CM-derived from various HCC cell lines (Mahlavu; Huh7; Hep3B) and primary HCC 26-1004 was analyzed by modified Boyden chamber assays. CM derived from control 10% DMEM was used as control. Data shown are averages of quadruplicate wells ± SEM; **p <0.01. **(Bi)** Representative immunofluorescence images and **(Bii)** number of migrated cells of CM-Dil labeled MSC (white arrows) or MRC5 in HCC 26-1004 at Day 1 and 4 post intraperitoneal injection into the mice. Nuclei were counterstained by DAPI. Figure presented at x200 original magnification. (**C** and **D**) Representative flow cytometric dot plot and histogram of EpCAM surface expression on **(Ci)** Huh7 cells alone, **(Cii)** HCC 26-1004 and **(D)** Huh7 and MSC. Respective isotypic controls are included as indicated.

The migratory activity of MSC toward HCC was evaluated in a modified Boyden chamber *in vitro*. We observed that MSC displayed significant migration towards conditioned medium (CM) derived from various HCC cell lines (Mahlavu, Huh7, Hep3B) and primary HCC cultured from patient-derived xenografts (denoted as HCC 26-1004) when compared to control medium (Figure 1A). However, human lung fibroblast cells, MRC5 which served as controls, did not exhibit significant migratory activities to the same CM. In addition, MSC also did not migrate towards CM derived from normal human fibroblast cells (data not shown). The abilities of these MSC to migrate toward HCC tumors were subsequently confirmed following intraperitoneal injection of either CM-DiI-labeled MSC or MRC5 into HCC 26-1004 (Figure 1Bi). We observed a significantly higher number of CM-DiI-labeled MSC versus MRC-5 in the tumor at both day 1 and 4 (Figure 1Bii), suggesting that the MSC could target specifically to HCC *in vivo*.

### Activation of EpCAM signaling is involved in MSC recruitment to HCC

**Figure 2:**
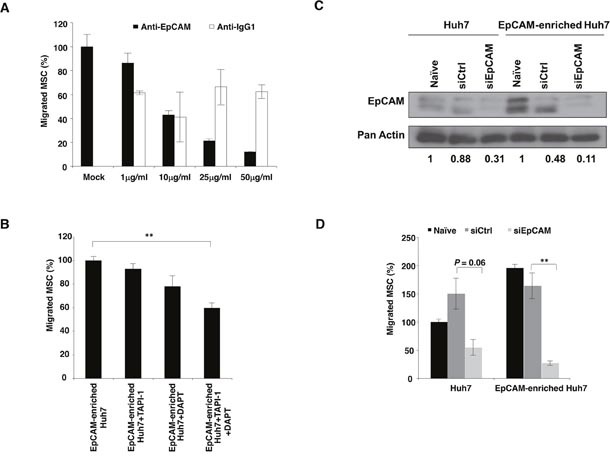
Activation of EpCAM signaling is involved in MSC recruitment to HCC Migration of MSC toward **(A)** CM-derived from Huh7 in the presence of either EpEx (BerEP4) or mouse IgG1 antibodies; **(B)** CM-derived from EpCAM-enriched Huh7 cells was analyzed in the presence of TAPI-1, DAPT or a combination of both inhibitors for 24 hours. Effect of EpCAM knockdown in Huh7 and EpCAM-enriched Huh7 cells was examined by **(C)** EpCAM protein expression. Pan actin served as a loading control. **(D)** Migration of MSC towards Huh7-CM or EpCAM-enriched Huh7-CM transfected with siCtrl or siEpCAM was determined. Naïve untransfected cells were used as control. All data are presented as mean ± SEM from at least three independent experiments; **p<0.01.

In an attempt to study the possible relationship between EpCAM and MSC recruitment to HCC, surface expression of EpCAM on MSC and tumor cells were first examined by FACS analysis using FITC-conjugated EpCAM antibodies. High levels of EpCAM expression could be detected in representative HCC cell line (˜59.90%; Huh7 cells; Figure 1Ci, as well as, Hep3B and Mahlavu cells supplementary Figure 1Eii-1Eiii respectively) and primary cells cultured from human HCC tumor biopsy (˜70.24%; HCC 26-1004 cells; Figure 1Cii). Neither MSC nor MRC5 express detectable levels of EpCAM (Figure 1D and Supplementary Figure 1D and 1Ei respectively). Next, we ask whether the migration of MSC towards HCC-derived CM could be inhibited in the presence of increasing concentration of antibodies against EpCAM. The percentage of MSC migration was observed to be 100 ± 10 for parental cells; and 87 ± 8; 43 ± 4; 21 ± 2.5; 12 ± 0.7 were scored in the presence of 1 μg/ml; 10 μg/ml; 25 μg/ml and 50 μg/ml of antibodies against EpCAM respectively (Figure 2A). This was not observed when anti-IgG antibodies were used. To investigate whether migration of MSC towards EpCAM-expressing HCC requires the activation of EpCAM signaling, the migratory activities of MSC towards FACS-enriched EpCAM Huh7 cells were examined. Combined treatment with TAPI-1 and DAPT prevented EpCAM shedding and subsequent nuclear appearance of EpICD. Thus, most of EpCAM expression was localized to the plasma membrane in the treated groups while both cytoplasmic and nuclear expressions of EpCAM proteins could be detected in the control group treated with DMSO vehicle (Supplementary Figure 2). This was accompanied by a significant reduction in the migratory activities of MSC towards EpCAM-enriched Huh7 cells in the presence of TAPI-1 and DAPT (Figure 2B). There was a notable reduction, albeit not statistically significant, in MSC migration in single treatment groups. Together, these results indicate that tumor tropism of MSC is influenced by the activation of EpCAM signaling pathway through the enzymatic activities. To further confirm the role of EpCAM in modulating the migrating potential of MSC, siRNA against EpCAM was performed and compared in EpCAM-enriched versus parental Huh7 cells. The two different bands represent the glycosylated (˜ 40 KDa) and the basic isoforms (˜35 kDa) of EpCAM [13]. The efficiency of EpCAM knockdown was subsequently confirmed by western blot analysis (Figure 2C). The results demonstrated that the migration activities of MSC in CM derived from naïve EpCAM-enriched Huh7 doubled those of naïve Huh7 as expected (Figure 2D; black box). In the presence of siEpCAM, migration of MSC towards CM derived from both cell types was impaired when compared to either siCtrl or naïve. The decrease in MSC migration was significant between siEpCAM versus siCtrl EpCAM-enriched cells (*p* = 0.008) but not between siEpCAM versus siCtrl parental Huh7 cells (*p* = 0.06). Together, these findings indicate that the migration of MSC is indeed mediated through the EpCAM associated signaling event.

### MSC migrates to EpICD ^high^-expressing HCC

**Figure 3:**
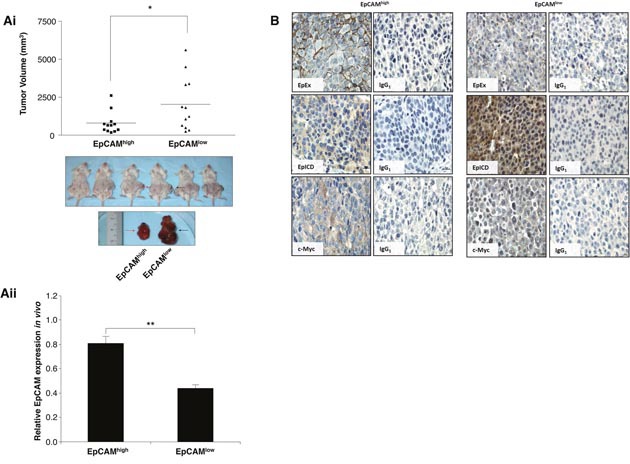
Activated EpCAM in HCC confers oncogenicity **(Ai)** Mean tumor volumes of FACS-sorted EpCAM^high^ (■) and EpCAM^low^ (▲) Huh7 cells injected subcutaneously into NODSCID mice in the right (red arrow) and left flanks (black arrow) respectively; *p<0.05. Bottom panel, the inset shows representative tumors at the end of the study (arrows). **(Aii)** Quantitative real-time PCR was performed on EpCAM^high^ and EpCAM^low^ tumors at the beginning of tumor formation. Relative EpCAM expression levels were normalized to 18S and plotted. Data is represented as mean of triplicates ± SEM; **p<0.01. **(B)** Representative images of EpEx (BerEP4), EpICD and c-Myc immunohistochemical staining in EpCAM^high^ and EpCAM^low^ tumors. Respective isotypic controls are included as indicated. Data shown are averages of triplicate samples ± SEM **p<0.01.

To elucidate the role of EpCAM in MSC migration, we examine the abilities of MSC to migrate towards NOD/SCID mice that have been serially transplanted with EpCAM^high^ versus EpCAM^low^ tumor xenografts. Contrary to our expectation, the tumor volumes derived from EpCAM^low^ were significantly larger than those of EpCAM^high^ of the same mouse (black versus red arrows respectively; Figure 3Ai). The basal levels of EpCAM mRNA expressions in representative animals were confirmed by real-time PCR analysis at the beginning of the tumor development (Figure 3Aii).

We next ask whether these EpCAM^low^ tumors may be low with respect to cell-surface epitope but contained higher fractions of the cleaved EpICD proteins compared to EpCAM^high^ tumors. Because EpCAM is activated through regulated intra-membrane proteolysis, high levels of surface EpCAM expression typically correspond to low proteolytic activities and thus, low expression of intracellular domain of EpCAM and vice versa. Immunohistochemistry studies performed on representative animals at end point using antibodies specific to the extracellular and intracellular domains of EpCAM (EpEx and EpICD respectively). The results showed that distinct membrane staining could be detected in EpCAM^high^ but not in EpCAM^low^ tumors (Figure 3B). In contrast, strong nuclei stains could be detected using antibodies directed against EpICD in EpCAM^low^ tumors, whereas the staining was diffuse and cytoplasmic in EpCAM^high^ tumors. The enhanced EpICD in EpCAM^low^ tumors also corresponded to an increase in the level of c-Myc protein expression, particularly in the nuclei whereas c-myc immunoreactivity is located predominantly in the cytoplasm of EpCAM^high^ tumors (Figure 3B). The modest increase in the level of c-Myc expression was also confirmed by real-time PCR analysis (Supplementary Figure 3A).

**Figure 4:**
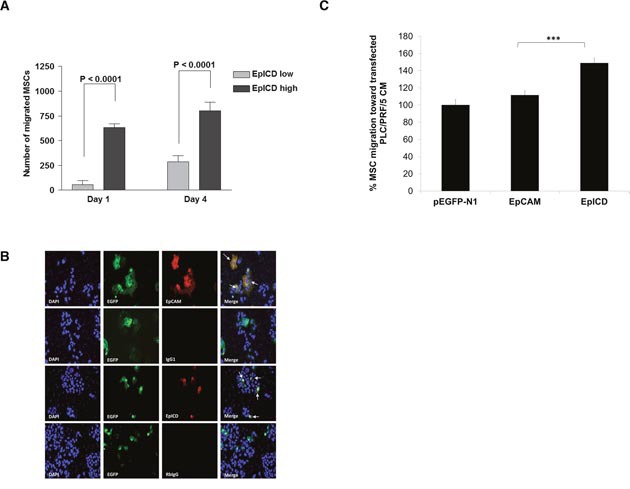
MSC migrates to EpICD^high^-expressing HCC **(A)** CM-DiI labeled MSC was intraperitoneally introduced into mice bearing bilateral tumors consisting of EpICD^low^ and EpICD^high^ in each animal. CM-DiI positive MSC cells within tumors of representative mice were scored at x200 magnification. Results are represented as means ± SEM; ***p<0.0001. **(B)** The expression of EpCAM and EpICD in pEGFP-N1-empty, EpCAM or EpICD transfected into PLC/PRF/5 was determined by immunofluorescence staining: EGFP (green); EpCAM or EpICD (red); nuclei were counterstained with DAPI (blue). White arrows indicate colocalization of either EpCAM or EpICD with EGFP-positive cells. Data were acquired under x200 original magnification using Nikon 90i Eclipse wide field microscope. **(C)** Migration of MSC towards CM derived from EpCAM or EpICD-transfected PLC/PRF/5 cells. Vector pEGFP-N1-transfected cells were used as control. The number of MSC migrated were normalized to that of vector-transfected cells and expressed as percent. Data shown are averages of quadruplicates ± SEM; *p<0.05

Next, we sought to determine whether tumors with higher levels of EpICD and c-Myc will recruit more MSC. CM-Dil labeled MSC was intraperitoneally introduced into mice bearing bilateral tumors consisting of EpCAM^high^ (i.e. EpICD^low^) and EpCAM^low^ (i.e. EpICD^high^) in each animal. The results showed that EpICD^high^ tumors attracted more MSC when compared to EpICD^low^ tumors (Figure 4A). To further validate the recruitment of MSC to highly oncogenic EpICD cells, HCC cells deficient in EpCAM expression were used. PLC/PRF/5 and MHCC97H cells have been previously reported by others to lack EpCAM expression. This was confirmed by RT-PCR (Supplementary Figure 3B). Next, we transfected empty pEGFP-N1 vector, and the same vector overexpressing the full-length EpCAM and EpICD domain into PLC/PRF/5 and MHCC97H cells. Forty-eight hours post transfection, migration assay of MSC was performed using CM derived from the various transfected cells. Expression of EpCAM or EpICD in transfected PLC/PRF/5 cells were confirmed with antibodies that recognized the full length EpCAM or EpICD (marked by white arrows; Figure 4B). Similar immunostaining were observed in transfected MHCC97H (Supplementary Figure 4A). Using conditioned media derived from these transfected cells as chemoattractant in the Boyden chamber migration assay, MSC were shown to migrate significantly to either PLC/PRF/5 or MHCC97H-transfected with pEGFP-N1-EpICD vectors when compared to full-length EpCAM or controls (Figure 4C and Supplementary Figure 4B respectively). MSC migration towards pEGFP-N1-EpCAM transfected PLC/PRF/5 cells, though enhanced, was not significant when compared to pEGFP-N1 vector (Figure 4C). However, when the same experiment was performed using another vector system, we could observe significant elevated MSC migration towards EpCAM-transfected cells when compared to vector alone (Supplementary Figure 4C). Taken together, these results demonstrate that MSC preferentially migrate to EpICD^high^ expressing HCC cells.

### EpICD confers HCC oncogenicity

**Figure 5:**
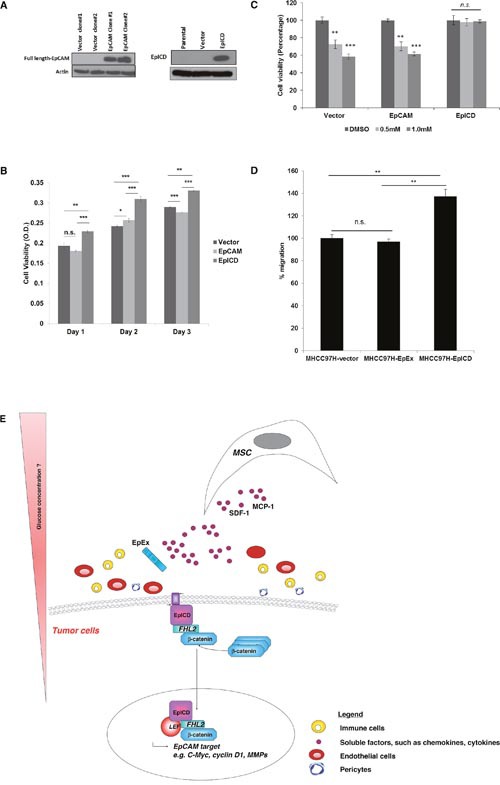
Enforced expression of EpICD confers oncogenicity in HCC **(A)** Reconstitution of EpCAM and EpICD in EpCAM-null MHCC97H cells, as analyzed by western blot. Cell proliferation of MHCC97H cells transfected with empty vector, full length EpCAM and EpICD was measured in **(B)** untreated cells at days 1, 2 and 3 post-transfection or at **(C)** 0.5 mM and 1.0 mM concentration of sorafenib, using DMSO as vehicle. **(D)** Migration of MSC towards CM derived from EpCAM or EpICD-transfected MHCC97H cells. Vector pEGFP-N1-transfected cells were used as control. The number of MSC migrated were normalized to that of vector-transfected cells and expressed as percent. Data shown are averages of quadruplicates ± SEM; *p<0.05. **(E)** A schematic illustration of MSC tumor tropism to EpCAM-expressing HCC cells.

Our findings showed that in the context of HCC, it is the activated EpCAM proteins i.e. EpICD that determine oncogenicity. This is in agreement with the previous report of Maetzel and colleagues, whereby human embryonic kidney cells (HEK293) expressing either full-length EpCAM or EpICD were demonstrated to confer oncogenicity in a SCID mouse model [14]. To further confirm this, stable clones expressing vector alone, EpCAM and EpICD were derived from EpCAM-null MHCC97H cells, and the expression was confirmed by western blot (Figure 5A). EpCAM-null MHCC97H cells expressing EpICD showed significant increase in cell proliferation (Figure 5B) and resistance against sorafenib (Figure 5C). Further, MSC showed enhanced migration activities toward conditioned media derived from EpICD but not EpEx, providing further evidence that MSC migrates to highly oncogenic tumor cells (Figure 5D). In this scenario, tumor cells with activated EpCAM i.e. the released EpICD associates with FHL2, β-catenin which form a nuclear complex with Lef-1 and induces transcription of genes associated with cell proliferation. (Figure 5E; Schematic diagram modified from Maetzel D et al., 2009) [14].

## DISCUSSION

In the present study, we demonstrated that MSC migration to HCC tumors can be effectively inhibited in the presence of TACE and ɣ-secretase inhibitors, which prevented the activation of EpCAM signaling event. Likewise, silencing the EpCAM expression through siRNA and antibody approaches also resulted in impaired MSC migration. Interestingly, animal studies showed that EpICD^high^ expressing HCC tumors, which represent a highly oncogenic tumor microenvironment, could recruit more MSC. This provides an additional benefit of using MSC as a cell-based carrier of therapeutic genes to target EpICD-enriched hepatic tumor cells.

In the field of cancer therapy, the most attractive feature of MSC is its inherent ability to migrate towards tumor cells or “a wound that never heals”. Migration of MSC to tumors has been demonstrated in many types of cancers, indicating that this phenomenon is independent of the type of tumor (reviewed by [15]). This attraction is mediated via paracrine signaling loop between the chemoattractants from the tumor microenvironment and the expression of the corresponding receptors in MSC or vice versa. For the first time, we demonstrated that it is the activated EpCAM i.e. the EpICD but not the cleaved soluble EpEx, that is important in mediating MSC migration to HCC cells.

EpCAM has been considered as putative hepatic stem cell marker because apart from its association with components of the Wnt pathway, it is also an essential factor in the maintenance of pluripotency in mouse and human embryonic stem cells. The maintenance of stemness is mediated through the ability of EpICD to regulate the promoters of reprogramming genes such as Oct4, c-Myc, Sox2, Nanog, and KLF4 [9]. Human embryonic kidney cells (HEK293) expressing either full-length EpCAM or EpICD confer oncogenicity in the SCID mouse model [14]. Since tumors are organized in a hierarchical manner similar to normal tissue with the exception of loss of control of tissue homeostasis, the above findings support the notion that EpCAM-positive tumor cells are capable of functioning as tumor-initiating cells [16–20]. Our results demonstrated that EpICD^high^ –expressing Huh7 formed larger tumor mass in comparison to similar number of inoculated EpICD^low^ Huh7 cells in the same animal (Figure 3A and 3B). Majority of the c-myc expressions in EpCAM^high^ tumors belong to the cytoplasmic stained tumor sections whereas c-myc is predominantly observed in the nuclei of the cells in EpCAM^low^ tumors. This finding is coherent with the finding where nuclear translocation of EpICD could induce the promoter activation of c-Myc and hence the increase in nuclear c-Myc [21]. Proto-oncogenes, c-Myc, cyclin D_1_ are target genes of EpCAM signaling [12, 22]. Interestingly, the regulatory element of the EpCAM gene promoter are regulated by transcription factor 4 (Tcf4), a downstream effector of the Wnt pathway [23]. Thus, it is not surprising that cyclin D_1_ is, in turn, a target of the Wnt pathway [24]. In fact, MSC and MSC-CM is capable of activating Wnt signaling in cholangiocarcinoma cells by promoting the nuclear translocation of β-catenin, and upregulated Wnt target genes including MMPs family, cyclin D_1_ and c-Myc [25].

Most recently, insulin activates cell cycle regulators including cyclin D_1_ which was shown to suppress hepatic glucose production [26]. Low glucose microenvironment is favorable to the migratory activities of MSC [27]. Thus, it is tempting to speculate that the enforced expression of EpICD leads to elevated cyclin D_1_ expression, the suppressed glucose levels together with other factors secreted by the tumor cells may create an attractive niche for MSC as illustrated in Figure 5E. In a recent study, IL-8, CXCL1-2-3/GRO and CCL2/MCP-1 have been implicated to promote MSC migration [28]. We have also demonstrated that enforce expression of EpICD in these EpCAM-null HCC cells could enhance cell proliferation (Figure 5B) and resistance against the standard of care drug for HCC, sorafenib (Figure 5C). Using a series of isogenic HCC cell lines, the results further showed that highly oncogenic HCC cells with activated EpCAM signaling recruit more MSC. Likewise, MSC migrates better towards Hep3B cells expressing high levels of endogenous EpCAM when compared to EpCAM-null MHCC97H (Supplementary Figure 4D). In a recent publication, EpICD was shown to serve as an oncogenic signal transducer in HCC by activating β-catenin, c-myc and cyclin D_1_ to promote cell proliferation [29]. At present, it is unclear why MSC migrates to pathological lesion tissues. Some studies have shown that MSC, especially those isolated from the sites of the tumors, aid in tumor cell proliferation, metastasis and the development of angiogenesis [30]. In contrast, we and others have demonstrated MSC could reduce HCC tumorigenesis [31, 32].

In summary, our results have shown that EpICD^high^ confers higher oncogenicity *in vivo*, with activated c-Myc expression and larger tumor burden in comparison with cells expressing lower fraction of EpICD. More importantly, MSC displayed differential homing potential toward tumor cells associated with higher oncogenicity and not the cleaved extracellular domain of EpCAM or HCC cells expressing low fraction of EpICD. This provides an additional benefit of using MSC as a cell-based carrier of therapeutic gene or imaging agent whereby specific targeting of the tumor initiating cells among the heterogenous nature of tumor mass and/metastasis scenario may be made possible, which is otherwise difficult to target by conventional approaches.

## MATERIALS AND METHODS

### Cell lines and cell culture

HCC cell lines PLC/PRF/5 and Hep3B were obtained from American Type Culture Collection (ATCC, Manassas, VA) while Huh7 cells were from Japanese Collection of Research Bioresources (JCRB cell bank, Osaka, Japan). Other HCC cells including Mahlavu and MHCC97H cells were kindly provided by Prof. Antoinette Lemoine (Inserm U1004, University Paris 11, France) and Prof Zhao-You Tang (Liver Cancer Institute, Fudan University, China) respectively. Lung primary fibroblast cell line MRC5 was a kind gift from Dr. Robert Chua (Duke NUS, Singapore). All cells were grown using standard tissue culture conditions. Primary cells derived from tumors were prepared by collagenase-dispase (Roche Applied Science, Mannheim, Germany) treatment of tumor samples into a single cell suspension. MSC7F3753 (denoted as MSC) were obtained from Lonza BioSciences (Walkersville, MD) and cultured in MSC growing medium (MSCGM) (Lonza BioSciences) for no more than 8 passages. Cell proliferation was determined at indicated time points by using CCK-8 kit (Dojindo Laboratories, Kumamoto, Japan) according to manufacturer's protocol.

### *In vitro* migration assay

Conditioned media (CM) was harvested from different cell types (1 × 10^6^ viable cells) at 48 hours post seeding. The harvested media was subsequently centrifuged at 1000rpm for 5 minutes, filtered through 0.2 μm filter to remove cellular debris and added to the bottom well of a modified Boyden chamber as previously described [33]. In brief, 1 × 10^4^ MSC were cultured in 24-well tissue culture insert on the upper well with an 8 μm pore size membrane (BD Biosciences). After 8 h, the number of propidium iodide-stained MSC nuclei was scored on the underside of the membrane under x 200 magnification at five random fields per replicate. For antibody blocking, Huh7 cells were treated with anti-EpCAM clone (EpEx) BerEP4 or normal mouse IgG1 (DAKO) at various concentrations overnight at 37°C prior to migration assay.

### Inhibitor and siRNA treatments

EpCAM-enriched Huh7 cells were treated with 40μM of TAPI-1 (Sigma-Aldrich) or 10μM of DAPT (Calbiochem, Merck KGaA, Darmstadt, Germany) or a combination of both inhibitors for 24 hours. Migration of MSC towards these inhibitors-treated CM was then analyzed. For EpCAM knockdown experiments, 1 × 10^5^ cells were cultured in a 6-well plate, and transfected with either siEpCAM or siCtrl at a final concentration of 100 nM using Lipofectamine RNAiMax (Invitrogen, Carlsbad, CA). After 72 hours post-transfection, cells were harvested and re-seeded at a density of 1 × 10^4^ cells in 24-well dish in 300 μl of 10% FBS containing DMEM medium. The western blot analysis and migration assays were performed as previously reported [33]. EpCAM-null MHCC97H cells expressing EpICD, EpCAM and Vector only were treated with 0.5mM or 1.0mM of Sorafenib (R01-900, SignalChem) for 24hr and the cells viability were determined using CCK-8 assay.

## SUPPLEMENTARY MATERIALS FIGURES


