# Supplementary material for: Human mesenchymal stem cells preferentially migrate toward highly oncogenic human hepatocellular carcinoma cells with activated EpCAM signaling
Source: Oncotarget. 2017 May 5;8(33):54629–39. doi: 10.18632/oncotarget.17633 (PMC5589609; doi:10.18632/oncotarget.17633)
Supplement: Supplementary file 1 [file oncotarget-08-54629-s001.pdf]

Human mesenchymal stem cells preferentially migrate toward highly oncogenic human hepatocellular carcinoma cells with activated EpCAM signaling

Supplementary Information

Online supplementary information contains supplementary materials and methods and figure legends.

SUPPLEMENTARY MATERIALS AND METHODS

Characterization of MSC

Immunohistochemistry staining and differentiation of MSC were performed as previously described [13].

Transfection and generation of stable clones

For transient transfection, MHCC97H or PLC/PRF/5 cells were transfected with pEGFP-N1-vector, pEGFP-N1-EpCAM, pEGFP-N1-EpICD or pCAG-3SIP-vector, pCAG-3SIP-EpCAM, pCAG-3SIP-EpICD plasmids using Lipofectamine 2000 (Invitrogen Life Technologies) following manufacturer's instructions. After 48 h, CM was harvested for *in vitro* migration assay. The cells were selected and maintained with 500 µg/ml of Geneticin (Sigma).

Immunoblotting

Expression of EpCAM or EpICD proteins was confirmed by immunoblotting assays. In brief, cells were lysed in protein lysis buffer (50 mM Tris, 150 mM NaCl, 1% Triton X-100) supplemented with HALT protease and phosphates inhibitor cocktail (Roche). Fifty micrograms of proteins were resolved by 10% SDS-PAGE and electroblotted onto PVDF membrane (Trans-Blot Transfer medium; Bio-Rad Laboratories). Membranes were blocked overnight with 5% non-fat milk in PBS containing 0.1% Tween 20 (0.1% PBT). Primary antibodies were diluted in blocking buffer and incubated at room temperature (RT) for 2 hours. Rabbit polyclonal EpCAM (1:250; C-10; Santa Cruz, CA), rabbit monoclonal antibodies against EpICD (1:500 dilution; 1144-1; Epitomics Burlingame, CA), and mouse monoclonal c-Myc (1:500; 9E10; Santa Cruz, CA) were ised. Following three 5-minute washes in 0.1% PBT, membranes were incubated with secondary antibody conjugated with horseradish peroxidase (goat anti-mouse IgG or goat anti-rabbit IgG) at 1:20 000 for 1 hour. After six 5-minute washes, membranes were exposed to enhanced chemiluminescence using Western Lightning chemiluminescent kit (Perkin-Elmer). Membrane was stripped and reprobed with pan-actin clone 5 (NeoMarkers) that was used as a loading control for each result. The band density of specific proteins was quantified using ImageJ (NIH) software.

Flow cytometry

Cells were harvested with 1 mM EDTA in PBS, washed twice with FACS buffer (0.1% sodium azide with 2% FBS in PBS) and resuspended in FACS buffer at $1x10^6$ cells per 100µL. 10µL of primary antibodies (Anti-Hu Epithelial Antigen-FITC (EpCAM)(Clone Ber-EP4)(Dako #F0860) or IgG controls per 100 µL of cell suspension were added and incubated for 30 min at 4°C in the dark. Cells were washed once with FACS buffer and surface expression was determined and acquired using FACSCalibur or FACSCantoII flow cytometer (Becton Dickinson, NJ). Data was analyzed for expression of PE or FITC using FlowJo software version 7.2.4 (Tree Star, Ashland, OR).

Total RNA isolation and real-time quantitative PCR

Total cellular RNA was isolated using RNeasy mini kit according to the manufacturer's instructions (QIAGEN, Valencia, CA). Total RNA from samples was reverse transcribed using Superscript™ Vilo Reverse Transcription kit (Invitrogen). Real-time PCR was performed on Rotor-Gene (QIAGEN) using following primers. EpCAM forward 5'-TTGCTCAAAGCTGGCTGCCAA-3' and reverse 5'-AGAGCCCGCTCTCATCGCAGT-3'; cMyc Forward 5'-TTGCTCAAAGCTGGCTGGCTGCCAA-3' and Reverse 5'-AGAGCCCGCTCTCATCGCAGT-3'; 18S forward 5'-CATGGCCGTTCTTAGTTGGT-3' and reverse 5'-GAACGCCACTTGTCCCTCTA-3'. Amplification was done under following conditions: 94°C for 15min; followed by 45 cycles of 94°C, 30 sec; 55°C, 30 sec and 72°C, 60 sec. Data was analyzed as relative gene expression utilizing ΔΔCt method, normalizing to Hep3B as positive control. Each sample was run in duplicates and at least three experiments were analyzed.

Immunofluorescence assay

Pre-labeled MSC or MRC5 (3 x 10^6 cells/500μL PBS) were injected intraperitoneally into tumor-bearing NODSCID mice. Tumors were harvested at day 1 and 4 post MSC injection, and frozen sections were prepared serially at 10μm (27 sections for a total of 2870-micron tumor thickness) and counterstained with 4', 6-Diamidine-2'-phenylindole dihydrochloride (DAPI; 1μg/mL; Roche Applied Science, Mannheim, Germany). Sections were mounted in glycerol (Invitrogen Life Technologies) and images examined and acquired using a Nikon Eclipse 90i upright microscope with CCD color digital camera using image acquisition software (NIS-elements AR 3.00). A total of 27 serial frozen sections at 100μm intervals were examined and the number of CM-DiI positive MSC were expressed as the total number of MSC per 2870 total microns.

Immunohistochemistry

Paraffin-embedded 5-μm thick sections of EpCAM tumor tissues were deparaffinized with xylene and rehydrated with graded ethanol. Antigen retrieval was performed in modified citrate buffer (DAKO). Endogenous peroxidase activity was quenched with 3% H_2O_2 in water for 10 minutes at RT. For detection of EpICD and c-Myc, sections were additionally blocked in 5% goat serum in PBS containing 0.1% tween-20 for 1 hour. Sections were then incubated with anti-EpCAM clone Ber-EP4 (5μg/mL; DAKO), anti-EpCAM clone E144 (EpICD) (0.396μg/mL; Epitomics) and anti-c-Myc clone 9E10 (10μg/mL; Santa Cruz) in antibody diluent (DAKO) for 30 minutes and 1 hour respectively. After respective washes, sections were subjected to anti-mouse and anti-rabbit HRP-conjugated polymer (EnVision kit; DAKO) for 30 minutes. Sections were finally developed with 3, 3-Diaminobenzidine (DAB) solution (EnVision kit; DAKO) and counterstained with Harris hematoxylin solution (Sigma Aldrich). Images were examined and acquired using image acquisition software (NIS-elements AR 3.00). Negative controls (i.e., isotype control) were included as indicated.

Primary HCC- and EpCAM-derived xenotransplantation models

All animal work was performed under guidelines approved by the National Cancer Centre Animal Care and Use Committee. Xenografts of patient-derived HCC line 26-1004 have been carried out as described previously [34]. To enrich for the EpCAM positive and negative cell population, FACS sorting was performed and 30% of the most EpCAM-positive (i.e. $EpCAM^{high}$) and EpCAM-negative (i.e. $EpCAM^{low}$) Huh7 cells were isolated and expanded. For generation EpCAM tumors, six to eight week old female NODSCID mice (n=12) from the same supplier were injected subcutaneously with FACS sorted $EpCAM^{high}$ and $EpCAM^{low}$ Huh7 cells (1x10^5) resuspended in a 1:1 mixture of PBS and Matrigel (BD Bioscience) in the right and left flanks respectively. Upon tumor uptake, $EpCAM^{high}$ and $EpCAM^{low}$ tumors were harvested, rinsed and non-viable portions trimmed off. Viable $EpCAM^{high}$ and $EpCAM^{low}$ tumors were weighed, minced and serially passaged in equal portions to the right and left flanks of a fresh batch of NODSCID mice respectively. Transplanted tumors were allowed to grow for two to three weeks. Tumor volume was calculated using the following formula: volume = 0.52 x length of tumor x (width of tumor) ^{2}.

Statistical analysis

Statistical analysis was performed using Prism 3.0 (Graphpad Software Inc., San Diego, CA). Nonpaired parametric data were compared with Student's t-test. P-value of 0.05 was considered statistically significant.

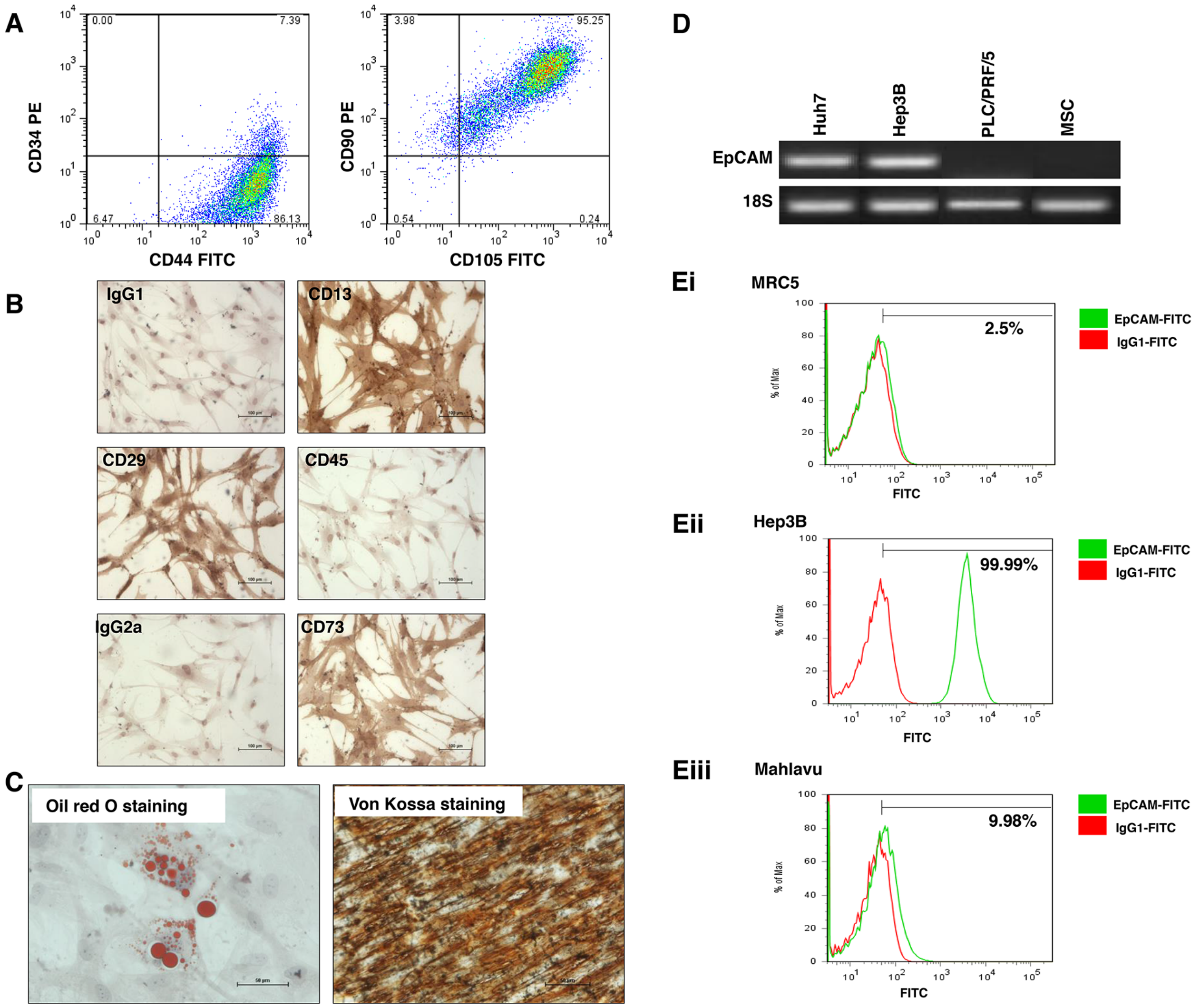

Supplementary Figure 1: Characterization of human bone marrow-derived MSC. **(A)** Representative flow cytometric dot plot for MSC stained with CD34 PE and CD44 FITC; CD90 PE and CD105 FITC. **(B)** MSC stained with CD13, CD29, CD45 and CD73. Respective isotypic controls are included as indicated. **(C)** MSC retained multi-lineage mesoderm differentiation potential as shown through Oil-Red O staining (adipocytes; left panel), and Von Kossa staining (osteoblasts; right panel). **(D)** RT-PCR was performed on total RNA isolated from HCC cell lines and MSC for expression of EpCAM levels. 18S was used as an internal control. **(E)** Flow cytometry analysis of surface EpCAM expression on **(i)** MRC5, **(ii)** Hep3B and **(iii)** Mahlavu cells. Cells were stained with Anti-Hu Epithelial Antigen-FITC (EpCAM) or isotype control mAb. Percentages of EpCAM-FITC positive cells in MRC5 (2.5%), Hep3B (99.99%) and Mahlavu (9.98%) were demonstrated in the flow cytometric histograms.

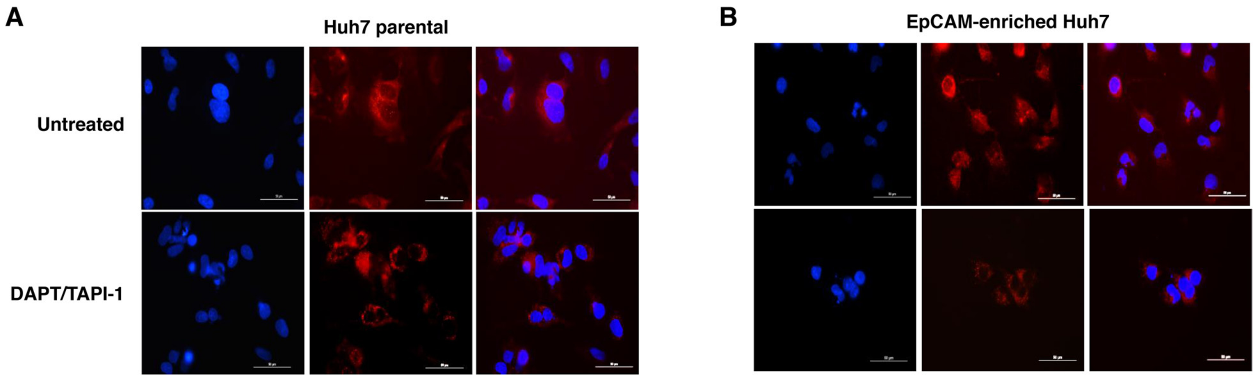

Supplementary Figure 2: Effect of TACE (TAPI-1) and γ-secretase inhibitors (DAPT) on subcellular localization of EpICD. Representative immunofluorescence images of EpICD on Huh7 cells treated with DMSO (control), DAPT; TAPI-1 or a combination of both inhibitors. EpICD (red); nuclei were counterstained with DAPI (blue). Subcellular localization of EpICD is indicated with yellow arrows.

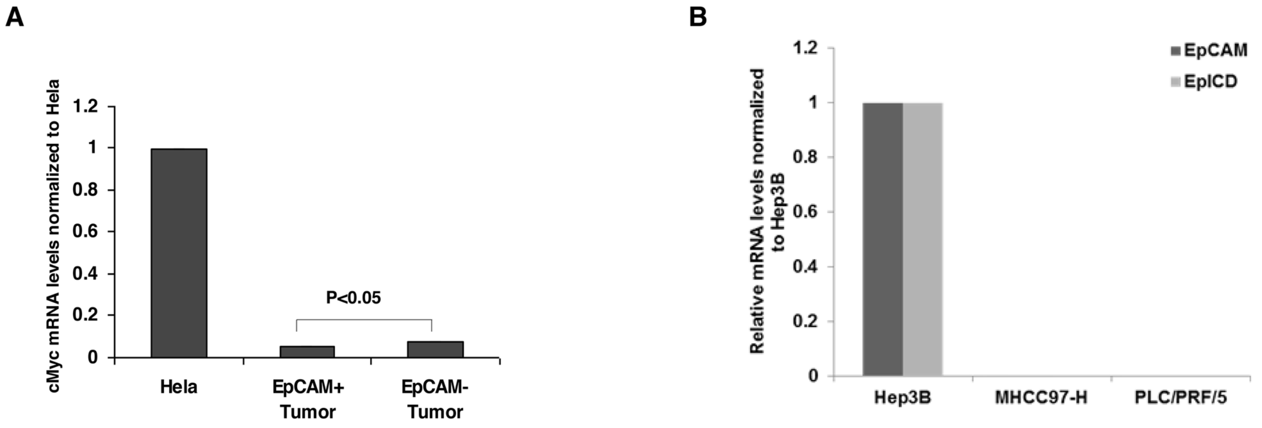

Supplementary Figure 3: C-Myc and EpCAM transcriptional expression in HCC cells. Quantitative RT-PCR against **(A)** c-Myc was performed on total RNA extracted from frozen tumors of EpCAM-positive (i.e. $EpCAM^{high}$) and EpCAM-negative (i.e. $EpCAM^{low}$) HCC tumors. **(B)** Two sets of primers were designed to confirm the lack of EpCAM expression in MHCC97H and PLC/PRF/5 cells. Hep3B served as positive control.

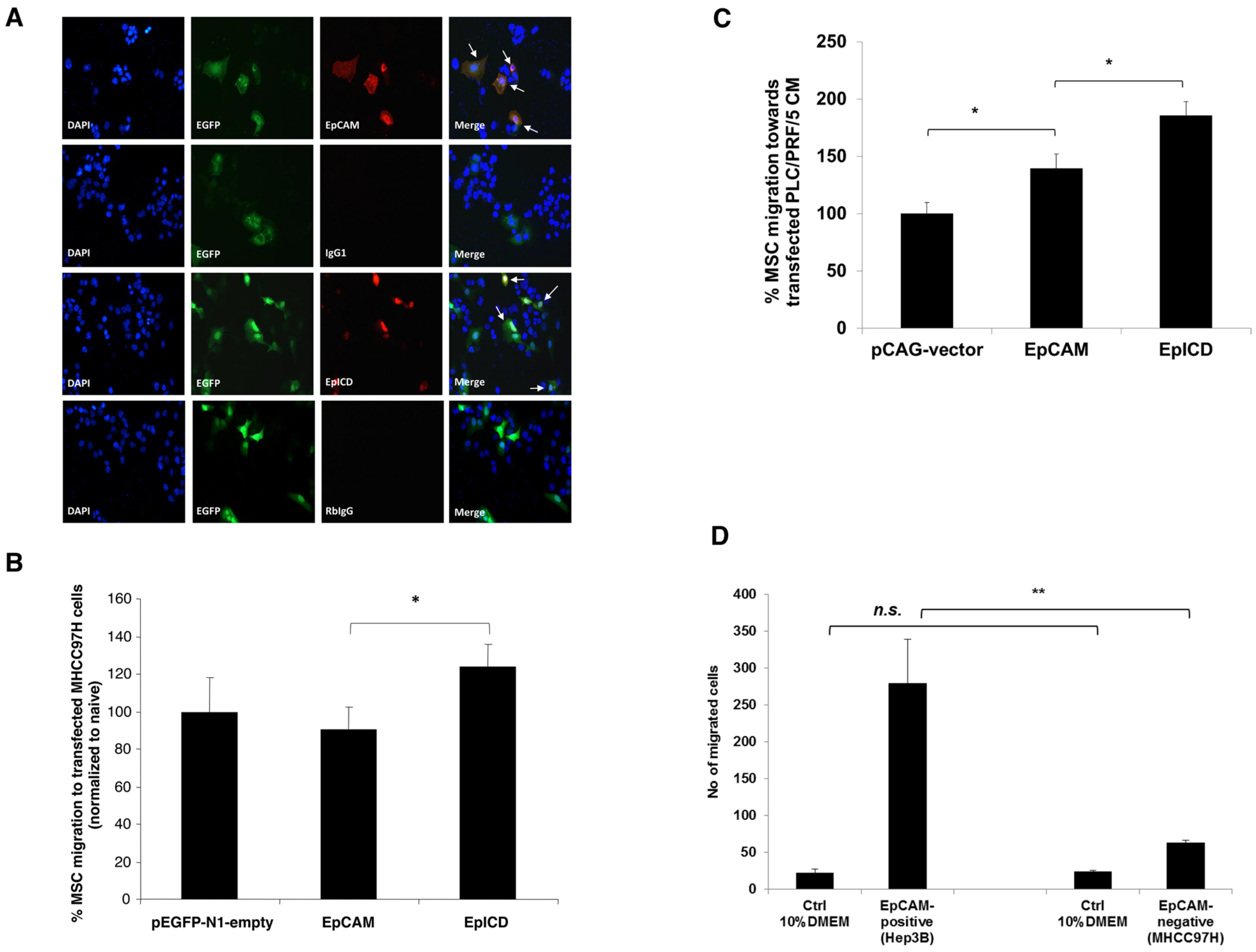

Supplementary Figure 4: MSC preferentially migrates towards EpICD enriched, but not EpEx, in HCC cells. **(A)** The expression of EpCAM and EpICD in pEGFP-N1-empty, EpCAM or EpICD transfected into MHCC97H was determined by immunofluorescence staining. EGFP (green); EpCAM or EpICD (red); nuclei were counterstained with DAPI (blue). White arrows indicate colocalization of either EpCAM or EpICD with EGFP-positive cells. Data were acquired under x200 original magnification. **(B)** Migration of MSC towards CM derived from EpCAM or EpICD-transfected MHCC97H cells. Vector pEGFP-N1-transfected cells were used as control. The number of MSC migrated were normalized to that of vector-transfected cells and expressed as percent. Data shown are averages of quadruplicates ± SEM; $^*p<0.05$. **(C)** Migration of MSC towards CM derived from EpCAM or EpICD-transfected PLC/PRF/5 cells. Vector pCAG-transfected cells were used as control. The number of MSC migrated were normalized to that of vector-transfected cells and expressed as percent. Data shown are averages of quadruplicates ± SEM; $^*p<0.05$. **(D)** Migration of MSC towards CM derived from EpCAM-positive Hep3B versus EpCAM-null MHCC97H. Data shown are averages of quadruplicates ± SEM; $^*p<0.05$
